# Inclusion of nicotine replacement therapy in the national essential drug list in India: Benediction for tobacco quitters?

**DOI:** 10.18332/tpc/156449

**Published:** 2022-12-08

**Authors:** Nancy Satpathy, Venkatarao Epari, Pratap K. Jena, Jugal Kishore

**Affiliations:** 1 Siksha 'O' Anusandhan Deemed to be University, Bhubaneswar, India; 2 KIIT School of Public Health, Kalinga Institute of Industrial Technology, Bhubaneswar, India; 3 Department Community Medicine, Vardhman Mahavir Medical College and Safdarjung Hospital, Delhi University, New Delhi, India

**Keywords:** nicotine replacement therapy, national essential medicines list, global adult tobacco survey, India

**Dear Editor,**

On 13 September 2022, the Government of India formally issued the National List of Essential Medicines (NLEM) with the inclusion of nicotine replacement therapy (NRT) for the first time^1^. This formal recognition paves the way for insurance coverage for NRT, supply/reimbursement in the public sector, and inclusion as a ‘must-know’ domain in medical–dental teaching^2^. More than half of the 99.5 million current adult smokers in India have a quit intention, two in five current smokers are already making a quit attempt, and seven in ten current smokers are making a quit attempt without assistance^3^. Thus with NRT, these smokers may have a 50–60% higher chance of quitting^4^, and there is evidence that the current limited NRT usage among current and former smokers^3^ may improve soon.

With an average smoking rate of 6.8 cigarettes per day (CPD) by a typical Indian daily smoker and the fact that 77.5% of the daily smokers are light smokers (CPD<10)^3^ for whom NRT has a limited effect^5^, this may raise serious concerns about the impact of NRT on tobacco cessation in the Indian context. Further, based on limited scientific evidence of NRT use among those using smokeless tobacco and smoking bidis, which are more prevalent forms of tobacco consumption than cigarettes in India^3^, the NRT alone as a pharmacotherapy may not change the cessation rate in India. In addition, the disparity in NRT use because of place of residence, gender and age, as reported in GATS-2, needs to be addressed.

The required scaling up of tobacco cessation interventions, following the reduction in tobacco cessation rates from GATS-1 (2009–2010)^6^ to GATS-2 (2016–2017)^3^, warrants the inclusion of additional pharmacotherapies (like bupropion, varenicline, etc.) in the NLEM and would benefit 199.3 million smokeless tobacco users. Creating enabling environments such as brief advice, anti-tobacco messages, and strict enforcement of tobacco control laws etc., to address light smokers and non-daily smokers, is also essential in the Indian context.

Though the addition of NRT to the NLEM represents an important milestone toward smoking cessation interventions in India, additional contextual modifications for effective treatment of smokeless tobacco users, bidi smokers, light smokers and occasional smokers need to be considered in the national tobacco control efforts.

## Data Availability

The data supporting this research are available from the authors on reasonable request.
